# Supplementary material for: The Construction and Exploration of a Comprehensive MicroRNA Centered Regulatory Network in Foxtail Millet (Setaria italica L.)
Source: Front Plant Sci. 2022 May 6;13:848474. doi: 10.3389/fpls.2022.848474 (PMC9121102; doi:10.3389/fpls.2022.848474)
Supplement: Supplementary file 1 [file Data_Sheet_1.zip › Sit-miR171i_Seita.1G262900.1_1082_TPlot.pdf]

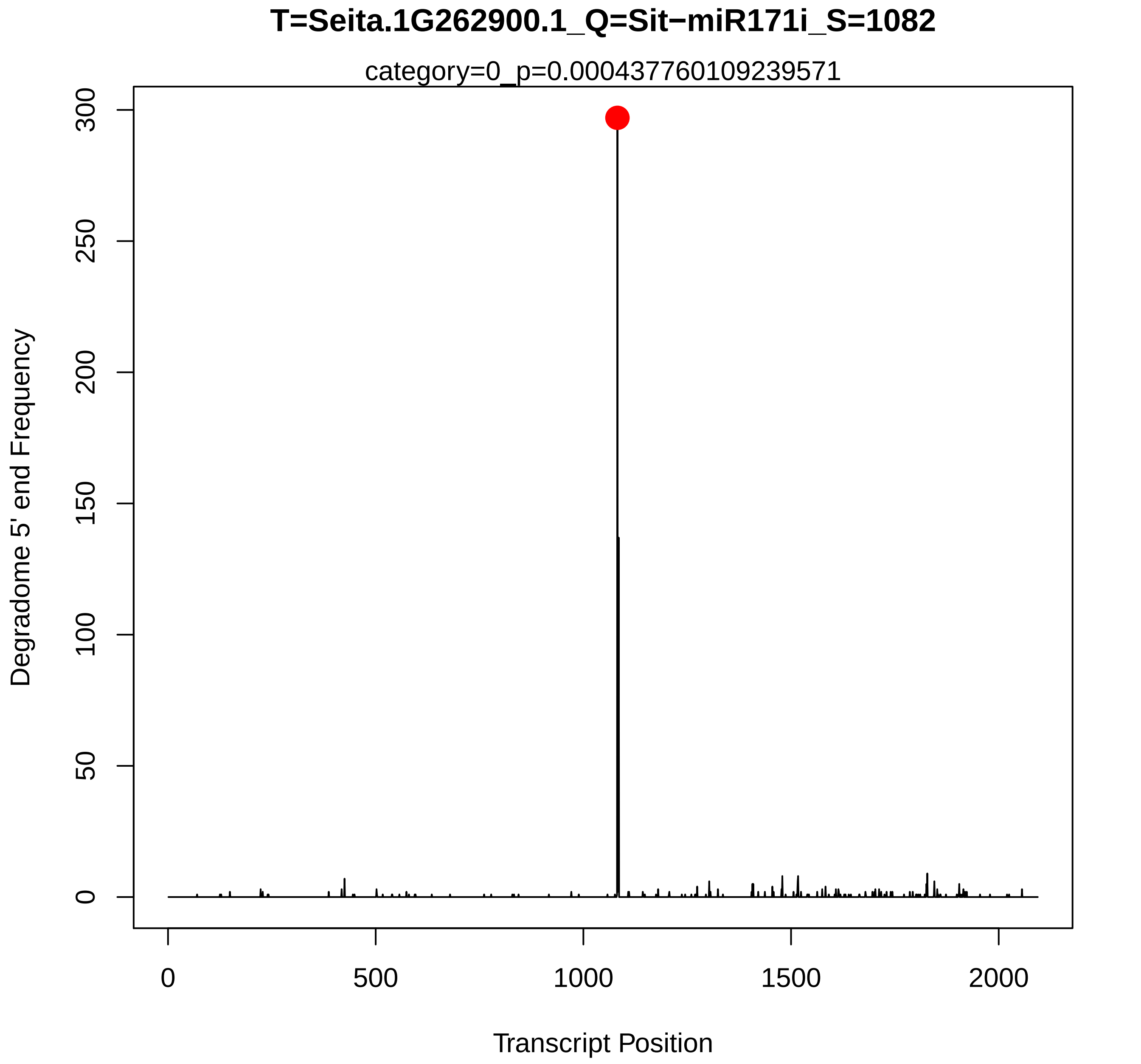

T=Seita.1G262900.1_Q=Sit−miR171i_S=1082
category=0_p=0.000437760109239571
Degradome 5' end Frequency
Transcript Position
0
50
100
150
200
250
300
0
500
1000
1500
2000
